# The family *Thermoactinomycetaceae*: an emerging microbial resource with high application value

**DOI:** 10.3389/fmicb.2025.1507902

**Published:** 2025-01-28

**Authors:** Mingmei Zhong, Zhenchun Sun, Chunhui Wei, Bertrand Muhoza, Haojie Tian, Maoqiang Liu, Shuyi Qiu, Dounan Li

**Affiliations:** ^1^College of Liquor and Food Engineering, Guizhou University, Guiyang, Guizhou, China; ^2^Guizhou Academy of Tobacco Science, Guiyang, Guizhou, China; ^3^Liquor Making Biological Technology and Application of Key Laboratory of Sichuan Province, Sichuan University of Science & Engineering, Yibin, Sichuan, China; ^4^School of Food Science and Technology, Jiangnan University, Wuxi, Jiangsu, China; ^5^Sichuan Langjiu Group Co. Ltd., Luzhou, Sichuan, China

**Keywords:** *Thermoactinomycetaceae*, underexplored sources, heat-resistant enzymes, bioactive compounds, environmental protection

## Abstract

In recent years, interest has increased in the use of microorganisms to obtain additional valuable resources for green and sustainable development. Preliminary functional analyses have indicated that members of the family *Thermoactinomycetaceae* have good application potential for the production of novel specific enzymes, high-value bioactive compounds, novel secondary metabolites and the promotion of efficient biomass conversion. Therefore, they can be considered a new class of microbial resources with potentially high value. However, the lack of culture and culture-independent techniques coupled with the uncertain taxonomic status of the family *Thermoactinomycetaceae*, has made exploring these potential applications challenging. This paper reviews the distribution characteristics and functional properties of the family *Thermoactinomycetaceae*, providing a detailed interpretation of the potential application value of this group and proposing a set of systematic resource development strategies based on a combination of culture-dependent and culture-independent strategies to exploit its potential for resource applications.

## 1 Introduction

The family *Thermoactinomycetaceae* refers to a group of chemoheterotrophic, gram-positive extremophilic microorganisms with white or yellow aerial hyphae (Seyed Shirazi and Hamedi, 2023). This group was first described in 1899 (Mo et al., 2023) and has always been considered a typical representative of microbial dark matter (MDM). Most members of this family exhibit good heat resistance, and some exhibit anaerobic and high salinity tolerance, like *Salinithrix halophila* isolated from the soil of the hypersaline wetland (Zarparvar et al., 2014). The family *Thermoactinomycetaceae* has been observed to inhabit various environments, including diverse soil types (Jin et al., 2022), high-temperature compost (Mironov et al., 2021), oceans (Wang et al., 2019), and hot springs (Ming et al., 2017). In recent years, the rise of metagenomics has enabled researchers to confirm the existence of this family in more environments, like traditional food fermentation systems (Hu et al., 2020; Ling et al., 2022; Mu et al., 2023; Pang et al., 2023; Xi et al., 2022).

At present, investigations of the functional properties of the family *Thermoactinomycetaceae* are still in their infancy. Studies on existing strains have demonstrated their great potential for applications in the production of novel heat-resistant enzymes, and high-value bioactive compounds (Jin et al., 2022; Lomthong et al., 2016; Shimizu et al., 2014). Moreover, the family *Thermoactinomycetaceae* can also affect the microecological environment through metabolism and play a special role in the ecological cycle process. For instance, during the thermophilic phase of natural high-temperature composting, this group can predominate and play a crucial role in enhancing composting effectiveness (Yu et al., 2018, 2015). The deep exploitation of these resources in the future will support modern enzyme engineering, food processing, agriculture, environmental protection, and other industries.

However, the limitations in research technology and the absence of systematic strategies have emerged as significant challenges in realizing the full exploitation of the potential application value of the family *Thermoactinomycetaceae*. In this paper, we review the distribution characteristics and discuss in detail the enormous application value of them. In reference to the recent development of rare microbial resources in desert soils (Li et al., 2023), this paper proposes a systematic strategy of “culture dependence + culture independence”, which provides an essential foundation for efficiently exploiting the future application value of the family *Thermoactinomycetaceae*.

## 2 Taxonomic status of the family *Thermoactinomycetaceae*

The family *Thermoactinomycetaceae* was first discovered in 1899 by Tsilinsky, and phenotypically, this group of bacteria can form mycelial structures similar to those of *Actinobacteria*. As a result, it was initially placed in the class *Actinomycetaceae* as a separate genus, *Thermoactinomyces* sp. Over the next 100 years, researchers identified 13 different species at this taxonomic level based on morphological and some physiological characteristics. However, the overall small differences in phenotypic and physiological characteristics between members of the genus *Thermoactinomyces* have led to persistent controversy over the taxonomic status of the group. To resolve this situation, researchers have explored taxonom from the perspective of molecular biology. In 1981, researchers carried out the first taxonomic identification of the representative species of this genus based on 16S rRNA sequences (Cai et al., 2019), which showed that the genus *Thermoactinomyces* was genetically close to *Bacillus*; this marked the beginning of the identification of the genus *Thermoactinomyces* using molecular biology techniques (Li et al., 2002). By 2000, there were eight validly published species in the genus *Thermoactinomyces*, and then, based on phylogenetic analysis, the researchers proposed that the genus *Thermoactinomyces* should belong to *Bacillus* rather than *Actinobacteria*, and the eight species under the genus were revised to 6 (Yoon and Park, 2000). In subsequent studies, researchers found that it was still very difficult to accurately identify members of the genus *Thermoactinomyces* using only one set of criteria; the identification technique represented by polyphasic taxonomic identification provided a comprehensive new approach to solve the ambiguous taxonomic status of this genus. Based on the aggregation of taxonomic data and comprehensive determination, three new genera, *Laceyella* sp., *Thermoflavimicrobium* sp., and *Seinonella* sp. (Matsuo et al., 2006; Yoon et al., 2005) were identified based on the original *Thermoactinomyces* sp., and the taxonomic status of this group was redefined as follows: Domain *Bacteria*, Phylum *Firmicutes*, Class *Bacilli*, Order *Bacillales*, and Family *Thermoactinomycetaceae*.

**Figure 1:**
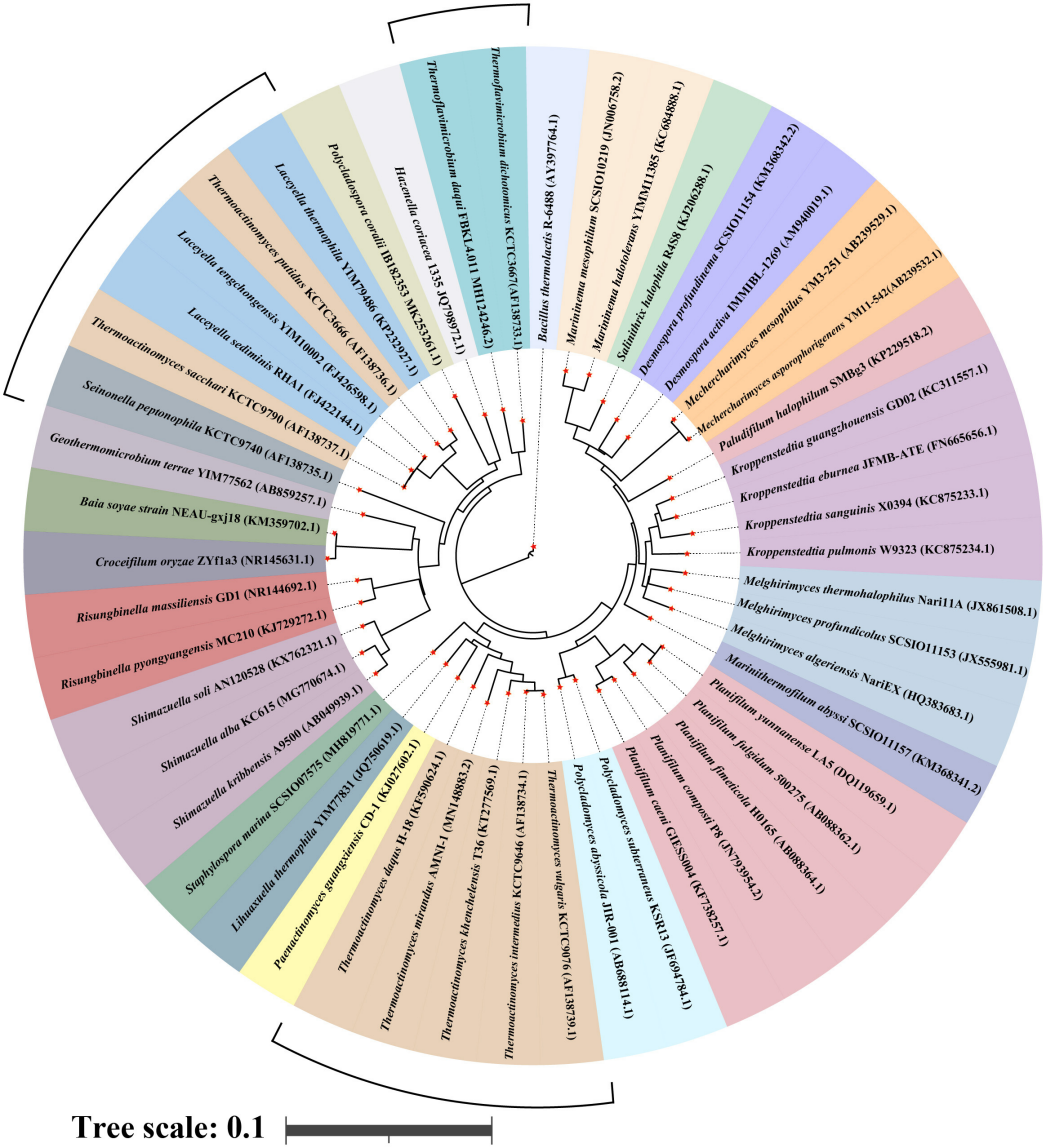
Cyclic phylogenetic tree of the family *Thermoactinomycetaceae* based on the 16S sRNA gene. Line segments indicate genera with ambiguous taxonomic status, including *Thermoactinomyces* sp., *Thermoflavimicrobium* sp., *Seinonella* sp., and *Laceyella* sp..

With the continuous development of multiphase taxonomic identification techniques, a set of more systematic, comprehensive, and accurate multiphase taxonomic identification techniques has been developed for *Thermoactinomycetaceae* based on morphological and structural characterization, analysis of cell wall components, analysis of carbon source utilization, determination of DNA G+C content, DNA–DNA hybridization (DDH), 16S rRNA gene sequence analysis, and whole-genome sequence comparison. These methods have further strengthened genotypic identification and emphasized the acquisition of strain genome-wide information and correct algorithms, replacing the original DDH with the overall genome-related index (OGRI) (Young and Bhally, 2017) as a more robust and objective gold standard for the determination of species (Chun et al., 2018; Ciufo et al., 2018). Supplemented with other methods, the establishment of OGRI has also opened the way for the development of a more robust and objective species identification method. The establishment of this method has led to a new round of research to clarify the taxonomic status of this group and to discover new species. The revision of the taxonomic status of four genera, i.e., *Thermoactinomyces* sp., *Thermoflavimicrobium* sp., *Seinonella* sp., and *Laceyella* sp. is the most prominent change (Jiang et al., 2019). The discovery of new species has entered a boom period, and the membership of the family *Thermoactinomycetaceae* continues to expand with the discovery of new genera such as *Staphylospora* sp. (Wang et al., 2019). To date, the family contains 24 genera and 47 species (Figure 1).

However, there are still limitations to the determination of the polyphasic taxonomy of the family *Thermoactinomycetaceae*, because the identification of environmental traces is not accurate and the lack of characteristic information in the existing gene database makes it difficult to achieve a precise comparison of genetic information between different species; all these problems need to be resolved by researchers through the future development of new identification tools. The family *Thermoactinomycetaceae* as an undetermined group between *Bacillus* and *Actinobacteria* has been observed to possess an mycelial structure that is similar to that of general actinomycetes. The mycelial differentiation mechanism varies notably among different actinomycetes. Consequently, the group of *Thermoactinomycetaceae* may also possess a relatively unique mycelial differentiation mechanism. In a recent study, Jiang Zhao et al. reported that the family *Thermoactinomycetaceae* has a mycelial differentiation mechanism that is independent of other *actinomycetes* and closely related to the spo series of genes (Jiang et al., 2019), which provides the possibility for accurate identification of the family and the discovery of more new members in the future.

## 3 Diversity of the family *Thermoactinomycetaceae* in various environments

**Figure 2:**
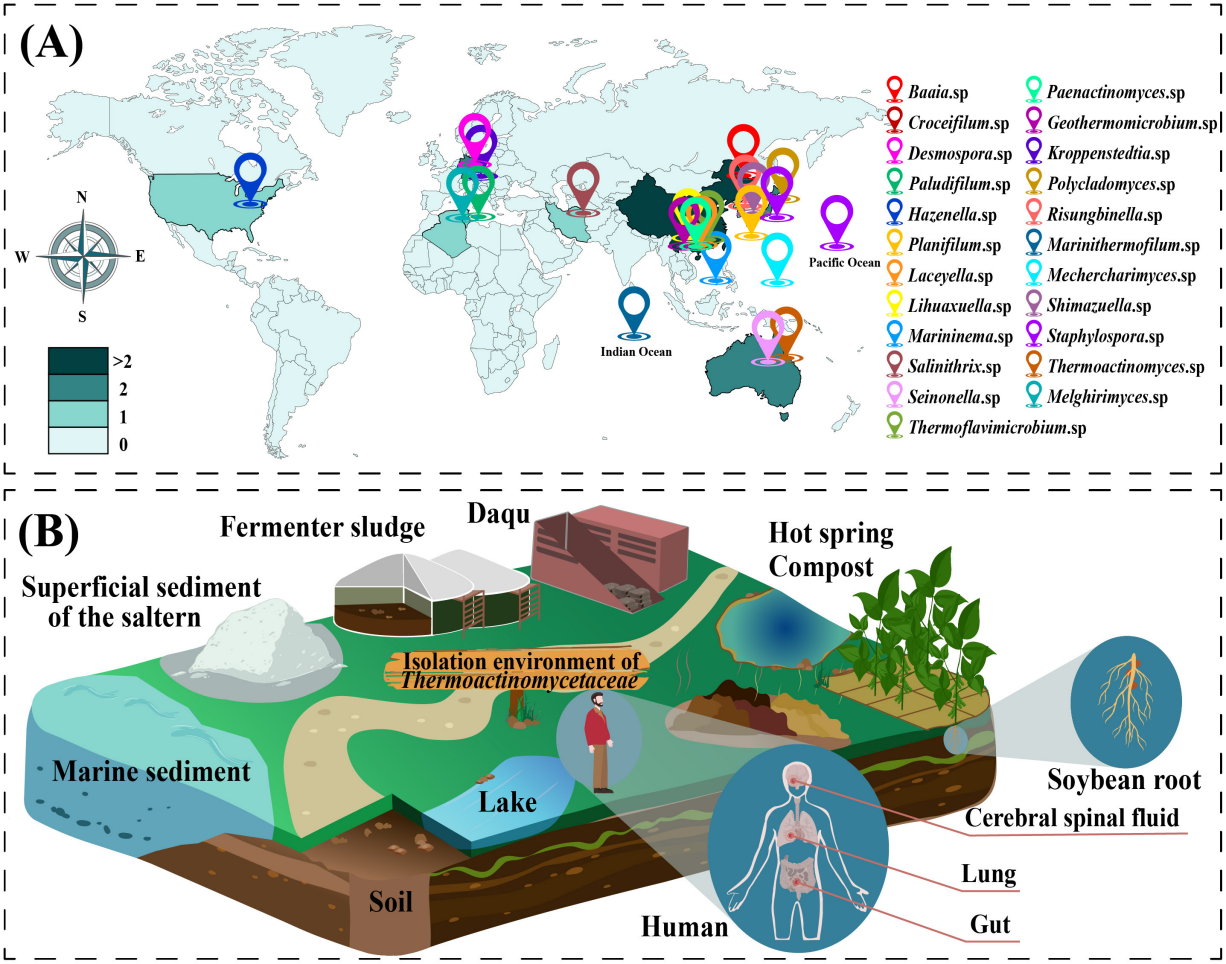
Distributional diversity of the family *Thermoactinomycetaceae*. **(A)** The global distribution of type strains of the family *Thermoactinomycetaceae*. A colored landmark indicates a genus of the family *Thermoactinomycetaceae*, and the landmark shows the geographic distribution location of the type species under this genus. **(B)** Diversity of growth environments in the family *Thermoactinomycetaceae*.

Members of the family *Thermoactinomycetaceae* are widely distributed in a variety of natural environments. Researchers have determined their distributions in various terrestrial environments on five continents. Figure 2A shows the global distribution of all model strains of this group that have achieved pure culture at the family level. The most extensive distribution is in Asia, mainly China and Japan. The family *Thermoactinomycetaceae* has also been confirmed to be present in seabed sediments in the Pacific and Indian Oceans.

The family *Thermoactinomycetaceae* is present in diverse growth environments, including in various soils, aquatic environments, high-temperature composts, animals, and plants (Jin et al., 2022; Mironov et al., 2021). Moreover, studies have confirmed that soybean roots (Guan et al., 2015), C-grade plastic surfaces (Von Jan et al., 2011) and seafloor sediments (Frikha-Dammak et al., 2016) can also be adequate habitats (Figure 2B). In recent years, with the development of techniques for detecting microbial diversity, the presence of this group has also been identified in traditional food fermentation environments (Dong et al., 2024; Ling et al., 2022; Zhu et al., 2018).

### 3.1 Soil

Soil has always been the optimal environment for isolating members of the family *Thermoactinomycetaceae*. Currently, soil samples account for the most significant proportion of all screening sources for pure cultured strains (Xian et al., 2015). As early as 2015, *Croceifilum oryzae* was isolated from rice paddy soil in Japan (Hatayama and Kuno, 2015). The off-white and mycelium-free strain of this family, which was subsequently designated as *Shimazuella soli*, was similarly isolated from farmland soil in South Korea (Jin et al., 2022). *Shimazuella kribbensis*, a mesophilic strain of this genus, was discovered in a soil sample collected from the mountains of South Korea (Park et al., 2007).

In addition to conventional soil environments, the family *Thermoactinomycetaceae* has also been isolated from extreme soil environments. For example, *Risungbinella pyongyangensis* (with a salinity tolerance of up to 8.0% w/v NaCl) was isolated from saline soil (Kim et al., 2015), and *Shimazuella alba*, an extremely drought-tolerant strain, was isolated from desert soil (Saygin et al., 2020). This again demonstrates the adaptability of the family *Thermoactinomycetaceae* to some extreme soil environments.

### 3.2 Water

Many strains of the family *Thermoactinomycetaceae* have been isolated from aqueous environments such as geothermal springs, marine lakes, and marine sediments. In the genus *Mechercharimyces*, both *Mechercharimyces mesophilus* and *Mechercharimyces asporophorigenens* were isolated from marine lakes (Matsuo et al., 2006). Moreover, the strains of this family isolated from seafloor sediments are even more diverse. In the South China Sea at a depth of 2,105 m and in the South Bay of Little Andaman Island, researchers screened two new strains of the family *Thermoactinomycetaceae*: *Marininema mesophilum* (Li et al., 2012) and *Marininema halotolerans* (Zhang et al., 2013). In 2015 and 2023, two novel type strains—*Marinithermofilum abyssi* and *Polycladospora coralii*—were isolated (Mo et al., 2023; Zhang et al., 2015).

In addition, the family *Thermoactinomycetaceae* is also widely distributed in extremely high-temperature volcanic and geothermal spring environments. Using culture-independent methods, researchers reported that *Thermoactinomyces* sp., *Laceyella* sp., and *Desmospora* sp. were abundantly distributed in these hot springs (Benammar et al., 2020; Mashzhan et al., 2021).

### 3.3 Compost

The first known representative from the family *Thermoactinomycetaceae* (*Thermoactinomyces vulgaris*) was isolated from decaying straw and manure used for composting (Bezuidt et al., 2016). Since then, a variety of strains have been found in high-temperature composts and sludge from biogas plants, and the most representative strain is *Planifilum* sp.. Recently, *Thermoactinomyces mirandus*, a new species of the genus *Thermoactinomyces*, has also been shown to be present in biogas plants (Mutschlechner et al., 2020). Moreover, by relying on modern culture-independent techniques, researchers have also reported that the family *Thermoactinomycetaceae* is the dominant microbial group in the thermophilic fermentation stage of hyperthermophilic composting (accounting for 29.9–36.1% of the total population) (Yu et al., 2015). With the extensive discovery of members of this family, their potential role as functional microorganisms for efficient biomass conversion during the fermentation stage of composting has been gradually revealed.

### 3.4 Traditional fermented foods

With the rapid development of metagenomics, the family *Thermoactinomycetaceae* has been confirmed to be widely distributed in traditional food fermentation systems. The most typical example of these foods is Daqu. Researchers have demonstrated the wide distribution of the family *Thermoactinomycetaceae* in various types of Daqu (Showing different aroma profiles), including soy sauce-flavor (Xia et al., 2022), Tao-flavor (Liu et al., 2023), sesame-flavor (Xie et al., 2020), and special flavor (Chen et al., 2021). In particular, the family *Thermoactinomycetaceae* is a dominant microbiota in high-temperature Daqu (Shang et al., 2022). Further analysis has shown that *Thermoactinomyces* sp. and *Kroppenstedtia* sp. were present in different types of high-temperature Daqu and were the main potential aromatic bacteria (Mu et al., 2023). Moreover, *Kroppenstedtia* sp. was also present and sustainable in distillers' grain during the stacking fermentation stage of the Jiang-flavor Baijiu, and it was considered to be closely correlated with key fermentation qualities of the distillers' grain *Kroppenstedtia* sp. is also present in fermentation systems such as vinegar and soybean paste, which is a core group in acetic acid fermentation (Zhu et al., 2018) and is associated with the production of flavors (free amino acids and aldehydes) (Ling et al., 2022).

The family *Thermoactinomycetaceae* is likely to play an essential role in promoting fermentation and determining product quality. Thus, it is a highly valuable resource for future exploration.

## 4 Potential applications of the family *Thermoactinomycetaceae*

**Figure 3:**
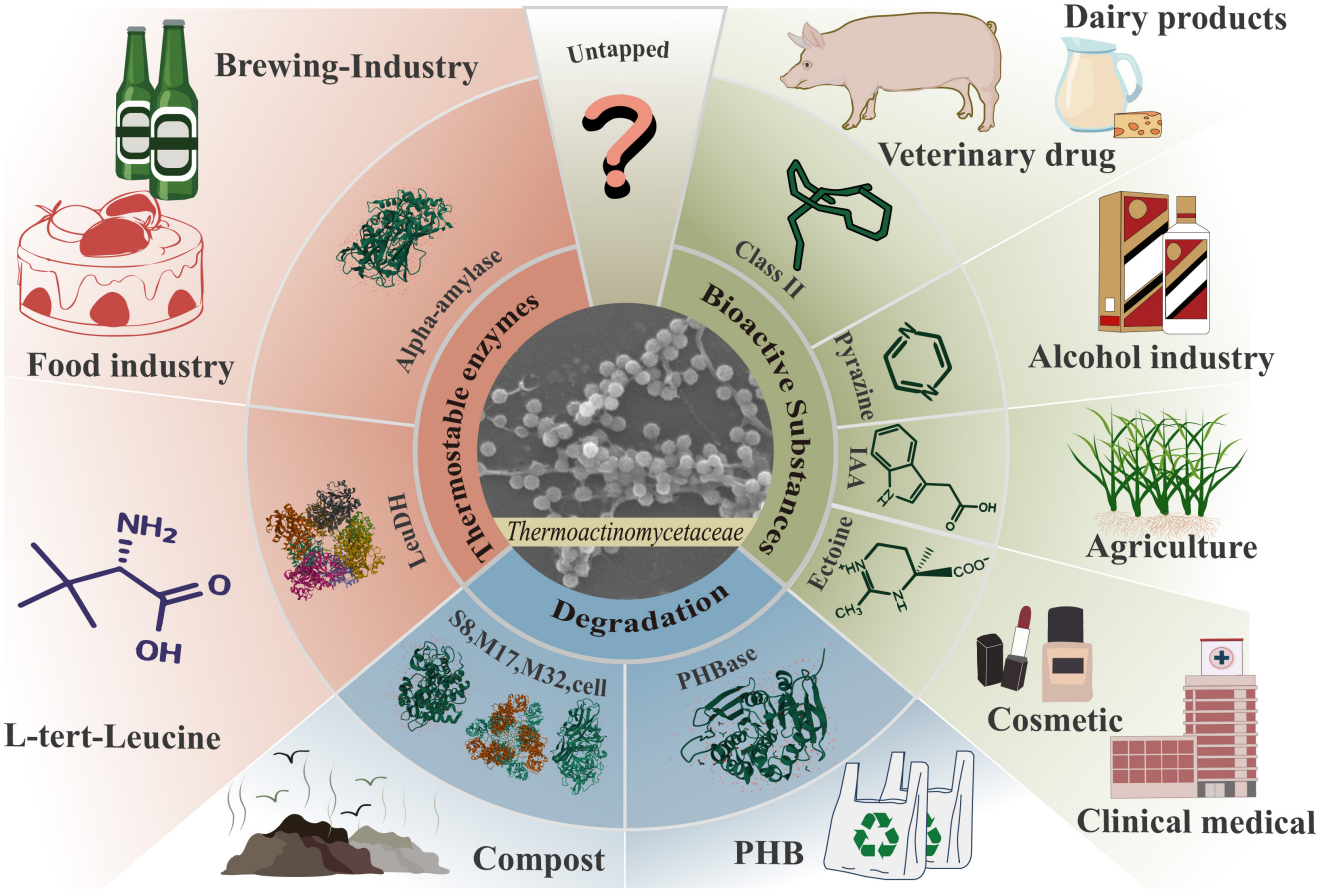
Exploiting the application potential of the family *Thermoactinomycetaceae*. The family *Thermoactinomycetaceae* has shown great potential for the production of thermostable enzymes, bioactive substances, and biomass degradation ability.

The family *Thermoactinomycetaceae* has long been recognized as a microbiota with significant potential for various applications. The initial focus was on the functional properties of metabolism-specific hydrolases (Shimizu et al., 2014), but research on the potential of environmental microorganisms for biomass conversion and environmental remediation has gradually increased (Zhang X. M. et al., 2021; Zhou et al., 2022; Zhu et al., 2021). In recent years, researchers have revealed significant potential in synthesizing bioactive and antimicrobial substances (Frikha-Dammak et al., 2020; Seyed Shirazi and Hamedi, 2023). Therefore, the family *Thermoactinomycetaceae* is considered an emerging microbial resource (Figure 3).

### 4.1 Novel heat-resistant enzymes

Heat-resistant enzymes exhibit good stability under extreme conditions such as high temperature, high acidity, and high pressure. This makes them suitable for a broader range of industrial applications (Kumar et al., 2019). The family *Thermoactinomycetaceae* adapts to high-temperature environments through the production of heat-resistant enzymes, making it a valuable source for the exploitation of novel heat-resistant enzymes (Table 1). The representative enzymes include the heat-resistant α-amylase family (El-Sayed et al., 2017; Shukla and Singh, 2015), heat-resistant M32 proteases (Zhang H. et al., 2021), subtilisin-like proteases (Ding et al., 2020), D-tagatose 3-epimerase (Guo et al., 2024), crude keratinase (Goda et al., 2021), chitinase (Shibasaki et al., 2014) and alkaline poly[(R)-3-hydroxybutyric acid] depolymerase (Thomas et al., 2022), current sources of which comprise the genera *Laceyella, Thermoactinomyces, Thermoflavimicrobium, Planifilum, Kroppenstedtia*, and *Lihuaxuella*. These enzymes all show good reactivity and structural stability above 65°C. This demonstrates that the family *Thermoactinomycetaceae* is a potential microbial resource for exploiting high-quality heat-resistant enzymes. Moreover, it can also serve as a valuable reference for improving the heat resistance of industrial enzymes. Targeted mutagenesis has been demonstrated to be an effective means of improving the heat resistance of industrial enzymes. It is therefore imperative to accurately identify the key mutation sites. Structural analysis of potential active sites (i.e., special amino acids, disulfide bonds, etc.) that confer good heat resistance to enzymes produced from the family *Thermoactinomycetaceae* can effectively help researchers to quickly identify the locations of key mutations in the same type of industrial enzymes that enhance their heat resistance.

**Table 1:** Example applications of the family *Thermoactinomycetaceae* in the synthesis of heat-resistant enzymes, bioactive substances, and efficient biomass conversion.

**Type**	**Product**	**Microorganisms**	**Application**	**Reference(s)**
Heat-resistant enzymes	α-amylase I, α-amylase II	*Thermoactinomyces vulgaris* R-47	Hydrolyzes pullulan and cyclodextrins	Shimura et al., 2011
	Low molecular weight α-amylase	*Laceyella* sp. RHA1A	Hydrolyzes amylose and amylopectin	Zhu et al., 2011
	α-amylase	*Laceyella sacchari* TSI-2	Efficiently removes starch stains from the cotton cloth.	Burhanoglu et al., 2020
	Raw starch degrading enzyme	*Laceyella sacchari* LP1P75	Produces cassava chip syrup As a substrate for bioethanol fermentation.	Lomthong et al., 2016
	Leucine dehydrogenase	*Laceyella sacchari*	Preparation of chiral pure L-tert-leucine	Wang et al., 2020
	β-1,3-Glucanase (LpGluA)	*Laceyella putida* JAM FM3001	Preparation of bio- logically active β-1,3-oligomers and protoplast of fungi and yeast cells and production of bioethanol from brown algae	Kobayashi et al., 2015
	D-lyxose isomerase(D-LI)	*Thermoflavimicrobium dichotomicum*	Preparation of functional sugars such as D-mannose, D-lyxose, and L-ribose.	Zhang et al., 2019
	Chitinase (Lp ChiA)	*Laceyella putida* JAM FM3001	Hydrolyzes colloidal chitin	Shibasaki et al., 2014
Efficient biomass transformation	An alkaline poly[(R)-3-hydroxybutyric acid] (PHB) depolymerase	*Lihuaxuella thermophila*	Hydrolyzes polylactic acid and polycaprolactone	Thomas et al., 2022
	Poly(L-lactide)-degrading enzyme	*Laceyella sucii* LP175	Degradation of bioplastic poly(lactide)/thermoplastic starch (PLA/TPS) blends.	Lomthong et al., 2017
	S8, M17, M32 proteases	*Paludifilum fulgidum*	Efficiently degrades nitrogen	Zhang H. et al., 2021
Bioactive substances	Lasso peptide	*Shimazuella soli, Lihuaxuella thermophila*	Resist bacteria and inhibit cancer cells from invading human lung cells, As a preservative for milk food and an antibacterial agent for veterinary drugs	Cao et al., 2021; Jin et al., 2022
	Pyrazines	*Laceyella sacchari* FBKL4.010	Perfuming substance, the functional factor of liquor. Dilates blood vessels, lowers blood pressure slightly, and prevents platelet aggregation and thrombosis.	Liu and Quan, 2024
	Indoleacetic acid (IAA)	*Thermoactinomyces vulgaris*	Plant growth regulators for agricultural production	Kedia and Singh, 2013
	Ectoine	*Paludifilum halophilum* DSM 102817^T^	Applications in bioprotection, cosmetics, pharmaceutical industry, biological waste, and wastewater treatment systems.	Ayadi et al., 2020
Antibiotics	Mechercharmycins A	*Thermoactinomyces* sp.YM3-251	It has a strong cytotoxicity	Lomthong et al., 2016
Anti-cancer agents	Urukthapelstatin A	*Mechercharimyces asporophorigenens*YM11-542	It inhibits the growth of human lung cancer A549 cells in a dose-dependent manner.	Matsuo et al., 2007

### 4.2 Efficient biomass transformation

Currently, the effective degradation of garbage and the efficient conversion of biomass are critical foci in sustainable development. The family *Thermoactinomycetaceae* is widely distributed in various waste composts and may play an important role as decomposers in the composting process. Therefore, exploring the potential of this taxon for efficient biomass conversion is worthwhile.

In recent years, researchers have discovered that *Laceyella thermophila* and *Laceyella sacchari* can rapidly degrade polymers such as poly-β-hydroxybutyrate (Thomas et al., 2022) and poly-L-lactic acid (Lomthong et al., 2017, 2022), processes which demonstrate this group has the potential for significant future contributions to the large-scale biorecycling of biodegradable plastics. Moreover, researchers have reported that *Planifilum* sp. is involved in the degradation/transformation of cellulose, hemicellulose, and lignocellulose in various composts (Xu et al., 2021). *Laceyella* sp. has also been shown to effectively increase the degradation and transformation of hydrocarbon pollutants in soil (Salam and Ishaq, 2019). This undoubtedly indicates the potential role of some strains of the family *Thermoactinomycetaceae* in the natural ecological cycle.

### 4.3 High-value bioactive substances

There are still significant market gaps today among high-value bioactive substances. The potential for synthesizing these substances by members of the family *Thermoactinomycetaceae* has been explored. Lasso peptides are natural peptide products (RiPPs) with solid stability and molecular scaffolding. Class II lasso peptides are equipped with resistance to microorganisms, peptide antagonists, and proteases, and have anticancer activities (Cheng and Hua, 2020). In 2022, a total of 15 novel genes encoding class II peptides were confirmed in *Shimazuella Soli* (Jin et al., 2022), which demonstrates the potential of the family *Thermoactinomycetaceae* as an essential source of class II lasso peptide synthesis.

Pyrazines (PYRs) are a class of high-value flavor components. As the investigation of the family *Thermoactinomycetaceae* has continued, the ability to synthesize pyrazines has been preliminarily demonstrated. *Kroppenstedtia* sp. and *Thermoactinomyces* sp. are positively associated with the synthesis of PYRs in high-temperature Daqu (Zhang Y. D. et al., 2021). In 2019, researchers further confirmed the ability of *Laceyella sacchari* to synthesize 2,5-dimethylpyrazine, trimethylpyrazine, and tetramethylpyrazine (Li et al., 2019, 2018). These findings indicate that some strains of the family *Thermoactinomycetaceae* have the ability to produce PYRs similar to those of *Bacillus* sp.. An in-depth investigation of the mechanism of pyrazine synthesis will be helpful for the development of the food processing, tobacco, and fragrance industries in the future.

### 4.4 Other potential functions

Many other potential functional values of the family *Thermoactinomycetaceae* have yet to be explored. One of its functions is as a potential microbial synthesis source for many novel antibiotics. In 2017, researchers demonstrated that crude extracts of *Paludifilum halophilum* inhibited the growth of pathogenic bacteria such as *Agrobacterium tumefaciens, Staphylococcus aureus, Salmonella enterica, Escherichia coli*, and *Pseudomonas aeruginosa* (Frikha-Dammak et al., 2020). Most recently, researchers identified several unknown UV-active compounds from crude extracts of *Laceyella* sp. and *Thermoactinomyces* sp. and tentatively determined that these substances have broad-spectrum antimicrobial activity (Seyed Shirazi and Hamedi, 2023).

## 5 Discussion of efficient exploration of the potential application value of *Thermoactinomycetaceae*

### 5.1 Current barriers

Researchers have long been searching for strategies to best exploit the resources of the family *Thermoactinomycetaceae* efficiently. There has been some progress, but overall, achievements have been limited. The main reasons for this situation are as follows: (1) Limitations of traditional culture-dependent technologies. At present, researchers do not have a deep understanding of the unique growth and metabolic characteristics of the family *Thermoactinomycetaceae* in different environments, so designing effective isolation strategies is challenging. Breaking the limitations of traditional methods and constructing an efficient strategy for obtaining pure strains are still basic issues. Based on the data obtained from culture-independent methods, it is necessary to further summarize the metabolic characteristics of the family *Thermoactinomycetaceae* in different habitats; this will be a key point allowing the continuous improvement of culture-dependent strategies. (2) Ambiguity of taxonomic status. The phenotypic similarities between different strains of the family *Thermoactinomycetaceae* have made them difficult to distinguish. With the development of modern molecular techniques, the accuracy of identification has dramatically improved. However, owing to the minimal sequence differences between strains, the accuracy of identification is still not ideal. (3) Shortages of culture-independent techniques. To date, there are still some limitations to this technology. First, it is impossible to detect trace species of the family *Thermoactinomycetaceae* in some environmental samples. Moreover, owing to limitations on extraction rate and sequencing depth, it is challenging to obtain all the data concerning potential functional genes, transcripts, and proteins (Lewis et al., 2020), which leads to incomplete functional prediction results for the family *Thermoactinomycetaceae*. In the future, it will be imperative to develop new culture-independent techniques that can provide direct functional evidence that can be applied to explore this resource.

### 5.2 Efficient ways to exploit potential value

The efficient exploitation of the potential application value of non-cultured microorganisms in the environment has posed a significant obstacle for researchers, no exception for the family *Thermoactinomycetaceae*. The main problem lies in the lack of a systematic strategy. The “culture-dependent + culture-independent” strategy was initially employed to investigate the complex microbiome within the human gut. The integration of the two approaches effectively addressed the limitations of employing these strategies individually. It allowed researchers to identify numerous functional microbial groups within the human gut which were previously considered “unculturable” This contributed remarkably to the efficiency of exploring the functional microbial resources within the gut (Lagier et al., 2018). Inspired by this idea, there is an increasing interest in utilizing this approach for the identification of uncultured extremophile microbial resources in the environment (Sood et al., 2021). In a recent exploration of uncultured bacterial groups in extreme desert environments, the use of this strategy resulted in the successful capture of a substantial number of novel bacterial group with potential applications that were not previously identified through direct sequencing (Li et al., 2023). This achievement has led to a significant expansion in the understanding of the microbiome present in the desert environment and also provides a significant reference for the effective exploitation of the family *Thermoactinomycetaceae* resources in various environments today.

**Figure 4:**
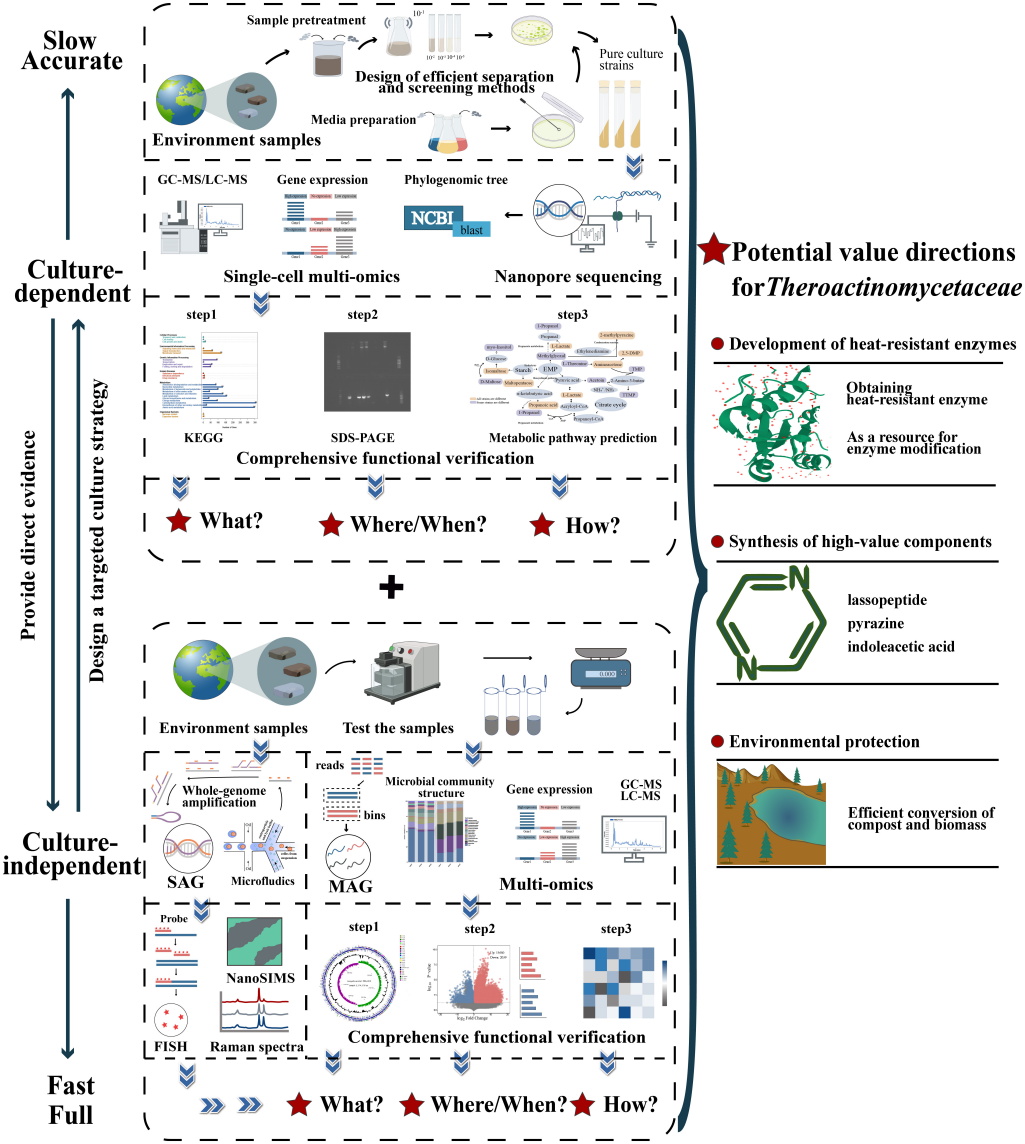
A systematic “culture-dependent + culture-independent” strategy to realize the full exploitation of potential resources of the family *Thermoactinomycetaceae*. The information obtained from culture-dependent methods can provide direct evidence of culture independence, and the information obtained from culture-dependent methods can be used for targeted culture strategies. The two methods complement each other, improve the accuracy and comprehensiveness of the database, and provide a way to efficiently exploit functional strain resources. Multiomics methods include genomics, transcriptomics, proteomics, and metabolomics. SAG, single amplification genome; MAG, metagenomes assemble genome; FISH, fluorescence *in situ* hybridization.

In this paper, the author proposes a combination of culture-dependent and culture-independent development strategies for the exploitation of the family *Thermoactinomycetaceae* resources (Figure 4). In this approach, we employ the culture-independent method to expeditiously identify members of the family *Thermoactinomycetaceae* from diverse environments. This enables the acquisition of expansive genetic information essential for taxonomic identification and functional development. Additionally, we can predict the growth and metabolic patterns of the taxon, thus providing crucial references for the development of a novel culture-dependent approach. The culture-dependent methods, such as culturomics, can collect a variety of genomic information through the acquisition of strains, which allows greater accuracy in confirming the functional potential of the family *Thermoactinomycetaceae* and a more intuitive exploration of their metabolic activities and potential ecological roles. Furthermore, strain acquisition can provide more open datasets, which can assist in the continuous upgrading of this technology.

#### 5.2.1 Constructing systematic culturomics

For the family *Thermoactinomycetaceae*, pure culture strains with potentially valuable functions can be obtained directly. Moreover, this approach has better accuracy in confirming the functional potential of a strain. However, several key points need to be noted.

##### 5.2.1.1 Design of efficient isolation methods

Establishing efficient isolation methods for strains is still essential for the construction of systematic culturomics. The most effective method is a targeted isolation strategy. Based on the assessment of growth and metabolic characteristics of the family *Thermoactinomycetaceae*, targeted adjustment of the composition of the medium and the modification of the culture conditions will improve screening efficiency. For the cultivation of microorganisms from estuarine sediment, the use of this strategy has led to the discovery of many new members (Kato et al., 2018; Pulschen et al., 2017; Wiegand et al., 2020). Moreover, given that some members of the family *Thermoactinomycetaceae* have close mutualistic relationships with other microorganisms, techniques such as cocultivation and *in situ* cultivation would be good choices. The effectiveness of these methods has been fully demonstrated in the isolation of new members of *Leucobacter* sp., and the excavation of commensal microorganisms in sponges (Bhuiyan et al., 2015; Jiang et al., 2016). Moreover, for strains of the family *Thermoactinomycetaceae* at the trace level, the recent emergence of microfluidic culture single-cell sorting culture technology is encouraging.

Notably, it is crucial to fully utilize the results of “multi-omics” analyses to guide the improvement of isolation strategies (Figure 4). The efficiency of culture-independent analysis allows researchers to quickly obtain predictions regarding the metabolism and functional properties of strains, which can lead to more targeted approaches to isolation. For example, the screening of dehalogenation microorganisms in industrial wastewater has been successful (Huang et al., 2023).

##### 5.2.1.2 Downstream technologies for resource development

Currently, with the development of chemical statistics, chemometrics, and other big data processing tools, genomics, transcriptomics, proteomics, and metabolomics have been integrated comprehensively to form a new “multi-omics” technology, which has also become a critical downstream technology for the development of strain resources (Palazzotto and Weber, 2018).

Analysis of the metabolic functional characteristics of pure cultured strains of *Thermoactinomyces* as well as the application of “multi-omics” analyses at the strain level are also important. In recent years, researchers have successfully confirmed the ability of *Sphingobacterium* sp. to biodegrade tetracycline through this method (Chen et al., 2023; Echegaray et al., 2023). However, very few reports exist utilizing a “multi-omics” analysis of the family *Thermoactinomycetaceae*. Although researchers have successfully predicted the potential of strains *Thermoactinomyces daqus* H-18 and *Laceyella sacchari* FBKL4.010 to synthesize flavor compounds at the genomic level (Li et al., 2019; Yao et al., 2015), this is inconsequential compared with the total potential functional resources of the family *Thermoactinomycetaceae*. For these reasons, the greatest challenge is the lack of accumulation and sharing of relevant datasets. A review of the current database also revealed that there are still limited multi-omics data and shared information for most members of the family *Thermoactinomycetaceae*. Therefore, a systematic “multi-omics” analysis of all pure culture strains of the family *Thermoactinomycetaceae* is essential. Through the acquisition and chemometric analysis of data, including genomic structure, functional annotation of coding genes/metabolic pathway prediction, transcription/protein translation levels under specific conditions, and metabolic profiling, the potential functional properties of the family *Thermoactinomycetaceae* in various habitats can be evaluated in depth by considering the following questions. “What?” (What kind of bacteria are present and what are the potential functional roles?), “Where/When?” (Where and when does the taxon play a functional role?) and “How?” (How do we perform its function?). Of course, addressing these questions requires the joint efforts of all researchers in this field.

#### 5.2.2 Novel culture-independent strategies

It is still imperative to rapidly predict the metabolic and functional characteristics of different strains of the family *Thermoactinomycetaceae* using a culture-independent perspective. The most representative method is 16S rRNA-based amplicon sequencing. Relying on this method, researchers have noted the presence of *Thermoactinomyces* sp., *Kroppenstedtia* sp., and other members of the family *Thermoactinomycetaceae* in vinegar, Daqu, and distillers' grains (Liu et al., 2023; Yang et al., 2023; Zhu et al., 2018; Zhuansun et al., 2022). However, the shortcomings of the technique's low resolution became apparent. Owing to the lack of genetic information, this method tends to overlook other potential functional properties.

With the emergence of new culture-independent analysis strategies such as meta-omics analysis and novel single-cell analysis, the shortcomings of amplicon sequencing technology can be remedied, which also provides considerable inspiration for future exploitation of this group (Figure 4).

##### 5.2.2.1 Meta-omics analysis

Since its inception, meta-omics analysis has rapidly become an essential tool for the in-depth analysis of MDMs in various environments and the discovery of uncultured microbial resources with potential applications (Knight et al., 2018). Currently, through the application of these strategies, researchers have identified a substantial number of unculturable Archaea taxa resources from snowfield soils. This achievement has yielded novel insights into the distribution structure of these groups and the patterns of their community functional properties in the context of climate change (D'Alò et al., 2023). Returning to the family *Thermoactinomycetaceae*, in the study of traditional Chinese Xiaoqu using the meta-omics strategy, researchers identified *Desmospora* sp. as closely associated with physicochemical indices and the production of crucial flavor metabolites of Xiaoqu (Zhao et al., 2021). This study provides a new understanding of the functional role of the family *Thermoactinomycetaceae* in fermentation systems and is also a good starting point for future in-depth exploitation of its application potential on a large scale using meta-omics analysis.

##### 5.2.2.2 Novel single-cell analysis

This technique can be classified into two types: analyses that rely on single-cell sorting and those that do not rely on single-cell sorting. For the first strategy, the critical point is a suitable cell sorting technology (Figure 4). Currently, with the development of flow cytometry, microfluidics, or micromodule integration, the efficient and rapid separation of single cells with different properties from biologically complex environmental samples has been achieved (Zhang et al., 2020).

Single-cell Raman spectroscopy-stable isotope coupling technology is a typical example. In a series of studies on nitrogen-fixing microorganisms, the advantages of using this technology have been well demonstrated (Cui et al., 2018; Tamamizu and Kumazaki, 2019). High-throughput sequencing technology continues to improve, and a novel single-cell multi-omics integration strategy is emerging. Consequently, it emerges as a favorable option for the exploration of uncultivated strain in ecological settings. However, it is worthwhile to consider the development of complementary technologies such as chemometrics and the integrated calculation of multi-omics data (Adossa et al., 2021).

Strategies that do not rely on single-cell sorting, such as single-cell fluorescence *in situ* hybridization-Nano secondary ion mass spectroscopy (FISH-NanoSIMS) analysis and single-cell NanoSIMS-stable isotope techniques, are also powerful tools. The use of these techniques has resulted in the discovery of novel cyanobacterial groups with nitrogen-fixing abilities in coastal and intertidal microbial mats (Woebken et al., 2015, 2012).

Although there are no reports on the application of the single-cell analysis strategy to the family *Thermoactinomycetaceae*, this strategy is highly promising. Within the environment, the same species of the family *Thermoactinomycetaceae* are likely to occur in different ecological locations, resulting in various cell differentiation effects. Thus, different cells of the same species have different metabolic properties. Such differences are often overlooked when using general meta-omics analysis, but the emergence of single-cell analysis can effectively address this challenge and help researchers to more fully and accurately interpret the ecological function diversity of the family *Thermoactinomycetaceae* in various environments.

### 5.3 Discussion of the direction of potential value exploration for this taxon

#### 5.3.1 Development of heat-resistant enzymes

Enzymes with heat-resistant properties are in great demand in industrial production. In order to maintain normal growth and metabolism, the family *Thermoactinomycetaceae* has large potential to synthesis various heat-resistant enzymes, and thus become an important microbial resource base for the discovery of novel heat-resistant enzymes (Yang et al., 2023). Meanwhile, how to develop the heat-resistant enzymatic potential based on the “culture-dependent + culture-independent” strategy in an orderly manner has become a new challenge for researchers.

In this paper, the following development strategy is proposed: when pure cultured strains are obtained, the utilization of these strains as enzyme-producing carriers enables the construction of a highly efficient, and low-cost enzyme-producing fermentation system through continuous optimization of fermentation conditions. This process leads to the direct and sustainable acquisition of high-value heat-resistant enzyme products following purification. This strategy has been successfully applied in cases like the efficient production of poly(l-lactide) (PLLA)-degrading enzymes and raw starch degrading enzymes (RSDE) by utilizing *Laceyella sacchari* as a carrier (Lomthong et al., 2022, 2020). The novel culturomics strategy is equally viable. This involves the use of single-strain level “multi-omics” analysis and chemometrics to effectively obtain the coding genes of heat-stabilized enzymes. Then, these enzymes can be obtained efficiently through the cloning of coding genes, the exogenous expression of engineered bacteria, and extraction and purification. By employing heterologous expression of a *AmyLa* α-amylase gene derived from *Laceyella* sp. DS3 in *Escherichia coli*, the researchers initially achieved large-scale production of the heat-stable α-amylase enzyme (Khoozani et al., 2019). In general, at the laboratory scale, the yield and purity of these enzymes are at a high level, and the basis for industrial-scale production is initially available.

Moreover, the acquisition of gene, transcriptome, and proteome data encoding heat-resistant enzymes from the family *Thermoactinomycetaceae* through culture-independent technique can also be utilized to provide important references for future heat stability modifications of industrial enzymes based on sequence, specific chemical bond targeted mutation and rigid structure enhancement (Dotsenko et al., 2020). When uncultivated members are present in the environment, meta-omics and single-amplified genomes (SAGs) can also rapidly identify genes encoding various heat-resistant enzymes (Sysoev et al., 2021) and increase the potential for heat-stable enzyme resources to be exploited on a larger scale.

#### 5.3.2 **Production of high-value components**

There are two approaches. The first is to conduct a deep exploration of pure cultured strain resources based on the results of the genome data. The functions of approximately 20-30% of the genes encoded by the genomes of strains in the family *Thermoactinomycetaceae* are unknown, and some of these will be closely linked to the metabolism of some potential novel high-value substances. Therefore, it is essential to analyse the functional roles of these unknown genes through single-strain-level “multi-omics” analysis guided by systematic culturomics. Class II lasso peptides have been demonstrated to exhibit notable biological activities. A putative novel class II Lasso Peptide consisting of 15 amino acids was identified through a systematic exploration of unknown genes from the genome of *Shimazuella* sp. (Jin et al., 2022). Furthermore, a thorough investigation of the genome of the salt-tolerant *Paludifilum halophilum* helps researchers successfully make a identification of the genes encoding key synthetic enzymes involved in tetrahydropyrimidine and streptomycin (Frikha-Dammak et al., 2021).

In situations when pure culture strains are not available, the culture-independent analysis of meta-omics at the single-cell level should also be considered. This technology allows the identification of a cell population with the ability to synthesize high-value substances as well as provides information about related genes, transcription, and protein expression, thus laying the foundation for the industrial production of these substances using engineered bacteria as carriers. In recent years, Single-cell transcriptomics has played an essential role in the characterization of the functional heterogeneity of the rumen microbiome (Jia et al., 2024). This also provides an example of its future use in the field of the family *Thermoactinomycetaceae*.

#### 5.3.3 **Environmental protection**

The family *Thermoactinomycetaceae* is the dominant microbiota during the thermophilic phase of high-temperature composting (Yu et al., 2018), which implies its potential to accelerate the decomposition of compost.

Obtaining culturable strains of the family *Thermoactinomycetaceae* directly from the complex microbiota in compost is challenging. It is more feasible to make breakthroughs through culture-independent analysis. The combined use of meta-omics, chemometrics, and new single-cell analyses allows rapid determination of the distribution of the family *Thermoactinomycetaceae* at different composting stages and the potential functional characteristics of each species. Meanwhile, the analysis of the culture-independent data can also provide support for the further optimization of high-temperature composting conditions. In the Tibetan Plateau, researchers identified the potential key microbiota like *Planifilum* sp. (belonging to the family of *Thermoactinomycetaceae*) in compost through culture-independent methods. Subsequently, based on analysis of omics data, researchers predicted the growth characteristics of these groups and formulated a more rational water replenishment strategy to continuously optimize the structure of these key microbiota in the compost. Ultimately, this strategy enhanced the maturation effect of the compost (Cai et al., 2022). A similar strategy was employed in the dairy manure composting process, where the enrichment of thermophilic microbiota (*Planifilum* sp.) by the heat pretreatment resulted in a significant increase in the lignocellulose degradation and humification efficiency of the compost in the actual situation (Zhang X. M. et al., 2021).

The large amount of data obtained by culture-independent analysis also enables researchers to clarify the mechanisms of compost transformation and the synergistic effects of the family *Thermoactinomycetaceae* and other microbiota during the composting phase. This really creates a foundation for the scientific assembly of artificial composting ecosystems (Zhang X. M. et al., 2021) and helps researchers gain a more comprehensive understanding of this group's growth profile in compost, which can provide guidance for the design of more targeted isolated methods for obtaining culturable strains that can contribute to compost maturation. In the longer term, this also offers significant materials for the future development of enhanced bacterial agents for high-temperature composting, based on these optimal strains.

In addition, some members belonging to the family *Thermoactinomycetaceae* also demonstrate a positive impact on ecological stability. In glacial environments, researchers have found that rare plants are more densely populated in areas where *Thermoflavimicrobium* sp. has a high distribution (Apple et al., 2019). A subsequent analysis of their ecological stabilization mechanism utilizing the strategy of “culture-dependent and culture-independent” is also of great practical significance.

## 6 Conclusions

As an uncultivated extremophile group in various natural environments, the family *Thermoactinomycetaceae* has shown great potential for the production of novel polarophilic enzymes, bioactive components, antibiotics, and the efficient conversion of biomass. The effective development of this group's potential resources has the capacity to generate new opportunities for the production of high-value biological products and to promote the healthy cycle of natural ecology in the future. However, the lack of clarity in the overall development strategy has resulted in the development of functional resources of this group remaining limited.

This paper offers a summary of recent investigative work by researchers exploring the potential application value of the family *Thermoactinomycetaceae*. Incorporating recent experiences aimed at cultivating uncultivated microbial resources in desert soils, we propose that the strategy of the “culture-dependent + culture-independent” strategy is regarded as a feasible scheme. This strategy employs the efficiency of culturing-independent techniques, including FISH and metagenomes assemble genome (MAG), to rapidly assess the potential applications of various targeted groups in the family *Thermoactinomycetaceae*. It also provides essential data support for improving the culture-dependent method to obtain more functional strains. On the other hand, the improved culture-dependent techniques have the potential to obtain more beneficial strains and determine their biological functions more intuitively. Undoubtedly, the strategy is merely a preliminary framework, and its viability must be assessed through a substantial number of research practices in the future.
